# The Clinical Safety and Efficacy of Cytarabine and Daunorubicin Liposome (CPX‐351) in Acute Myeloid Leukemia Patients: A Systematic Review

**DOI:** 10.1002/cnr2.70199

**Published:** 2025-05-09

**Authors:** Abdulwahab M. Alzahrani, Mohammed A. Alnuhait, Tariq Alqahtani

**Affiliations:** ^1^ Al Baha Health Cluster Ministry of Health Al Baha Saudi Arabia; ^2^ Pharmaceutical Practices Department College of Pharmacy, Umm Al‐Qura University Makkah Saudi Arabia; ^3^ Department of Pharmaceutical Sciences College of Pharmacy, King Saud Bin Abdulaziz University for Health Sciences Riyadh Saudi Arabia; ^4^ King Abdullah International Medical Research Center Riyadh Saudi Arabia

**Keywords:** acute myeloid leukemia, CPX‐351, efficacy, liposomal cytarabine/daunorubicin, relapsed/refractory AML, safety

## Abstract

**Background:**

Acute myeloid leukemia (AML) is an aggressive blood cancer with a poor prognosis when treated using conventional chemotherapy. CPX‐351, a liposomal formulation of cytarabine and daunorubicin, has demonstrated potential as a novel therapeutic strategy.

**Aim:**

This systematic review aims to evaluate the clinical safety and efficacy of CPX‐351 compared to standard induction chemotherapy in patients with AML.

**Methods:**

A systematic literature search was conducted using Web of Science, PubMed, Google Scholar, Ovid MEDLINE, and the Cochrane Library from 2011 to 2023. Overall, 14 clinical trials with more than 800 AML patients were included. Data on adverse events, survival outcomes, and response rates were extracted and analyzed.

**Results:**

CPX‐351 exhibited an adverse event profile that was comparable to that of conventional chemotherapy. It resulted in complete remission rates of 18%–41% in relapsed/refractory AML patients. For newly diagnosed individuals, CPX‐351 led to complete remission rates of 41%–58%, surpassing the 14%–40% associated with standard chemotherapy. Additionally, it extended overall survival by 3.6–5.7 months, with significant advantages noted in high‐risk cytogenetics and secondary AML cases.

**Conclusion:**

This review presents strong evidence supporting CPX‐351 as a therapeutic alternative with superior efficacy and comparable safety to standard chemotherapy across diverse AML populations. This represents a breakthrough in therapy, with demonstrated efficacy in AML cases.

**Abbreviations**
ACS: acute coronary syndrome
AE: adverse events
AkI: acute kidney injury
ALT: alanine transaminase
AML: acute myeloid leukemia
ARDS: acute respiratory distress syndrome
AST: aspartate aminotransferase
CDA: cytidine deaminase activity
CPX‐351: cytarabine and daunorubicin liposome
CR: complete remission
Cri: complete response with incomplete count recovery
DVT: deep vein thrombosis
ECOG: Eastern Cooperative Oncology Group
EFS: event‐free survival
FDA: Food and Drug Administration
FLAG‐Ida: fludarabine, cytarabine, G‐CSF, and idarubicin
GIT: gastrointestinal tract
HSCT: hematopoietic stem cell transplantation
IV: intravenous
LVEF: left ventricular ejection fraction
MDS: myelodysplastic syndrome
OS: overall survival
PK: pharmacokinetics
QTcF: Fridericia's corrected QT interval
RFS: relapse‐free survival
sAML: secondary AML
tAML: therapy‐related AML
TEAE: treatment‐emergent adverse events
URTI: upper respiratory tract infection

## 1 Introduction

Acute myeloid leukemia (AML) is a heterogeneous hematological malignancy characterized by the clonal expansion of myeloid progenitors in the bone marrow and peripheral blood [1]. It is the most common acute leukemia in adults, with an incidence of 3–5 cases per 100 000 individuals annually [1, 2]. Despite advances in treatment strategies, AML remains a challenging disease with poor long‐term outcomes, particularly in older patients [2, 3]. The standard induction therapy for AML has long been the “7 + 3” regimen, consisting of 7 days of cytarabine and 3 days of an anthracycline, typically daunorubicin. While this approach achieves complete remission in 60%–80% of younger patients, the outcomes in older adults remain suboptimal, with remission rates of only 40%–60% [2, 3]. Furthermore, the high relapse rates and significant toxicities associated with conventional chemotherapy underscore the urgent need for more effective and less toxic treatment options.

Recent years have witnessed significant progress in understanding the molecular landscape of AML, leading to the development of targeted therapies and novel drug formulations. One such innovation is CPX‐351 (Vyxeos), a liposomal formulation of cytarabine and daunorubicin in a fixed 5:1 M ratio [4, 5, 6, 7]. This encapsulation strategy aims to enhance the synergistic effects of these agents while potentially reducing off‐target toxicities. The U.S. Food and Drug Administration (FDA) approved CPX‐351 in 2017 for the treatment of newly diagnosed therapy‐related AML (t‐AML) and AML with myelodysplasia‐related changes (AML‐MRC) [8, 9, 10, 11, 12]. This decision was based on a phase III clinical trial demonstrating improved overall survival compared to conventional “7 + 3” chemotherapy in high‐risk AML patients [4, 7]. Given the potential impact of CPX‐351 on AML treatment paradigms, a comprehensive evaluation of its safety and efficacy is warranted [13, 14, 15]. This systematic review aims to assess the rates and types of adverse events associated with CPX‐351 compared to conventional induction chemotherapy regimens, evaluate its effectiveness in terms of remission rates and survival outcomes, and examine its potential role in improving treatment outcomes for high‐risk AML patients, including those with t‐AML and AML‐MRC [16, 17, 18, 19, 20]. By synthesizing the available evidence from randomized controlled trials and observational studies, this review seeks to provide clinicians and researchers with a comprehensive understanding of CPX‐351's place in the evolving landscape of AML treatment.

## 2 Materials and Methods

### 2.1 Study Design

This systematic review adhered to the guidelines outlined in the Preferred Reporting Items for Systematic Reviews and Meta‐Analyses (PRISMA) statement. The methodology employed aligns with widely recognized and recommended practices for conducting systematic literature reviews in medical research.

### 2.2 Search Strategies

A comprehensive search was performed across major medical databases, including Web of Science, PubMed, Google Scholar, Ovid SP (Medline), and the Cochrane Library, for studies published between 2011 and 2023. The search strategy incorporated a combination of keywords, titles, and Medical Subject Headings (MeSH) terms:

[(CPX‐351 OR daunorubicin/cytarabine liposome) AND (safety OR toxicity OR adverse effects OR toxic potential) AND (AML OR Leukemias, Acute Myeloid OR Acute Myeloblastic Leukemia OR Leukemia Xylogenous OR Acute Myelocytic Leukemia OR Acute Nonlymphocytic Leukemia OR Acute Non‐lymphoblastic Leukemia)]

Additionally, references cited in relevant research articles were manually reviewed to identify studies that could contribute further to the analysis.

### 2.3 Study Selection Criteria

Studies were included in this review if they met the following eligibility criteria. Eligible studies were required to be published as full manuscripts and written in English. They had to provide detailed descriptions of the intervention of interest and report relevant clinical outcomes. Both randomized and non‐randomized clinical trials were considered, provided they focused on adult participants aged 18 years or older and specifically addressed patients with acute myeloid leukemia (AML).

Exclusion criteria were applied to studies that did not report outcomes pertinent to the clinical question or used research designs other than clinical trials. Studies published before 2011, those available only in abstract form, and those involving animal studies were excluded. Additionally, duplicate publications of studies already reviewed were not considered.

### 2.4 Screening and Selection of Studies

After completing the database search, titles and abstracts of all identified studies were independently screened using Rayyan software. Articles meeting the inclusion criteria were then evaluated for full‐text eligibility.

### 2.5 Data Extraction

Data were extracted using a standardized form developed in Microsoft Excel by the primary author and subsequently reviewed by co‐authors. The collected information included the first author's last name, year of publication, and study location. Details about the clinical trials, such as their status, allocation, intervention model, blinding, and duration, were also documented. Additionally, data on study endpoints (primary and secondary), objectives, inclusion and exclusion criteria, and patient characteristics—including ECOG performance status, sex, age, sample size, and disease stage—were gathered. Information regarding CPX‐351 treatment, such as the stage of treatment and follow‐up duration, was recorded. Clinical outcomes, including overall survival (OS), event‐free survival (EFS), relapse‐free survival (RFS), complete remission (CR), complete remission with incomplete hematologic recovery (CRi), partial response, and adverse events (AEs, Grades 1–5), were also extracted to comprehensively evaluate the studies.

### 2.6 Risk of Bias and Quality Assessment

The risk of bias (ROB) for randomized clinical trials was assessed using the Cochrane ROB tool. This evaluation encompassed five major domains that focused on various aspects of trial design, execution, and reporting, guided by “targeted questions” to identify potential bias. Based on the assessment, outcomes were categorized into three levels: low ROB, indicating minimal bias across all domains; some concerns, where concerns were identified in at least one domain; and high ROB, reflecting substantial bias in one or more domains.

For non‐randomized studies, the quality was evaluated using the ROBINS‐I tool, with findings summarized in Table 4 [29].

## 3 Results

### 3.1 Study Selection and Characteristics

**FIGURE 1:**
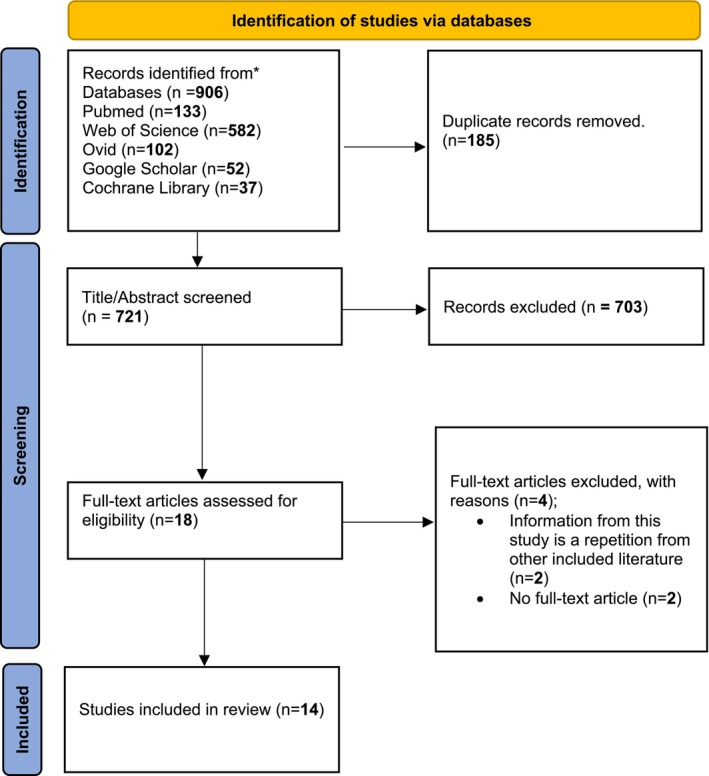
PRISMA flow diagram summarizing the study selection process, from 906 initial records to 14 studies included in the final review after screening and exclusions.

The process of study selection for this systematic review is illustrated in Figure 1 as a PRISMA flow diagram. An initial database search yielded a total of 906 records. Following the removal of 185 duplicate entries, 721 unique records were screened based on their titles and abstracts. Of these, 703 records were excluded as they did not meet the inclusion criteria. Subsequently, 18 full‐text articles were evaluated for eligibility. Four articles were excluded at this stage—two due to redundant information and two due to the unavailability of full‐text versions—resulting in the inclusion of 14 studies in the final systematic review [4, 19, 21, 22, 23, 24, 25, 26, 27, 28, 30, 31, 32, 33].

### 3.2 Included Studies and Participants

**FIGURE 2:**
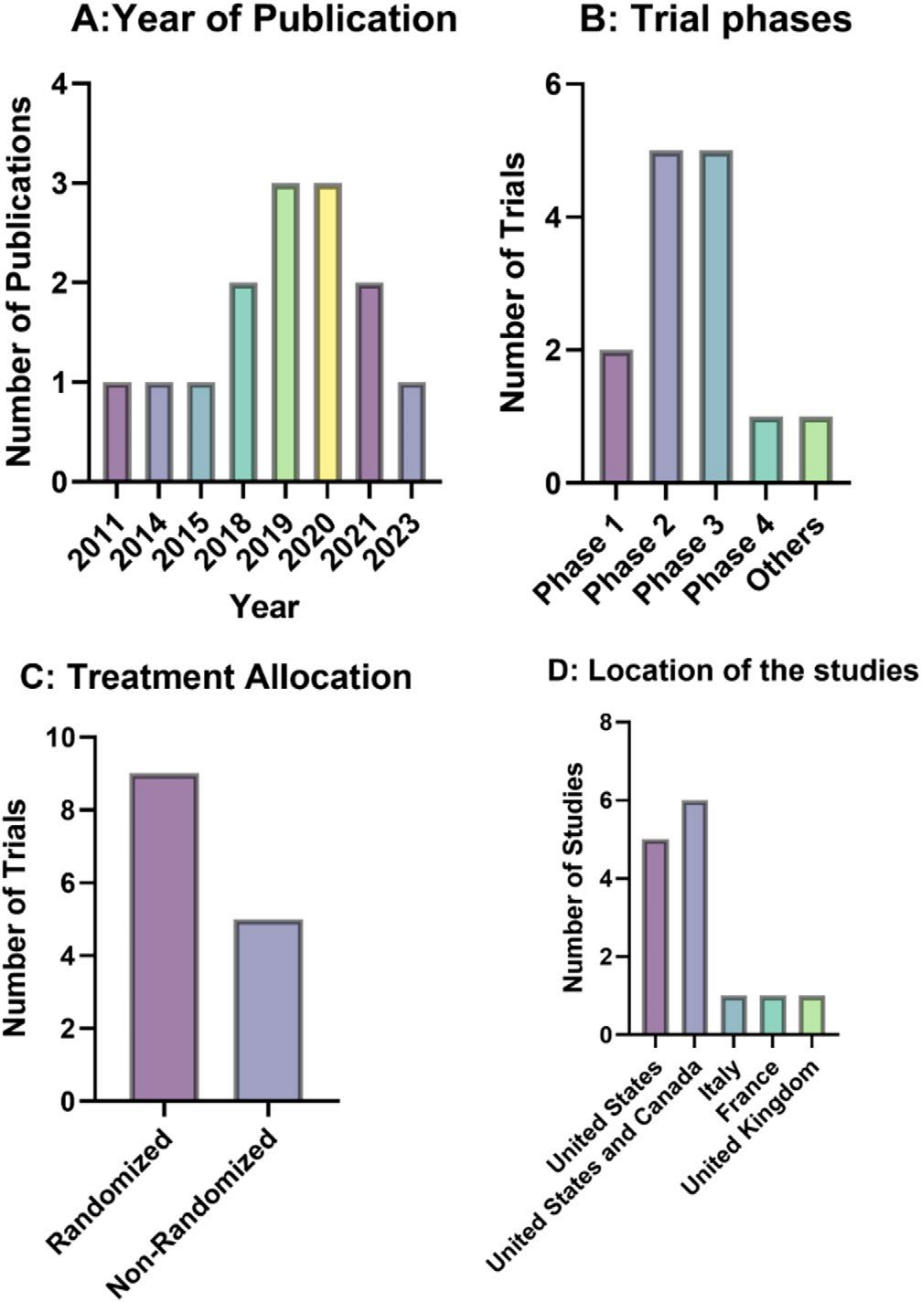
Overview of Clinical Trial Characteristics from 2011 to 2023. The figure summarizes clinical trial characteristics, including publication year trends (Panel A), trial phases (Panel B), randomization approaches (Panel C), and research locations (Panel D).

Figure 2 provides an overview of the characteristics of the 14 studies included in the systematic review, spanning publication years from 2011 to 2023. These studies were conducted in various locations, including the United States, Canada, Italy, and France, with the highest proportion published in 2019 and 2020 (21.43% each). The majority were phase 2 (35.71%) and phase 3 (35.71%) trials, employing randomized treatment allocation (64.29%) and a parallel assignment intervention model (64.29%). All studies were open‐label (100%), with none reported as unblinded. Sample sizes predominantly ranged from 51 to 250 participants (57.14%), while 28.57% included 1–50 participants. Geographically, most studies were conducted in the United States (42.86%), with fewer studies carried out in European countries, including Italy, France, and the United Kingdom.

Table 1 summarizes the essential details of the 14 clinical studies that evaluated the use of CPX‐351 for treating AML. The study designs included both single‐group and parallel‐arm comparisons. Specifically, five studies utilized single‐group designs to assess the safety, dosing, and preliminary efficacy of CPX‐351, while the other nine used randomized parallel‐arm designs to compare CPX‐351 to standard chemotherapy regimens such as the 7 + 3 treatment. The study phases ranged from early phase 1 studies with 10–48 patients to larger phase 2 and 3 studies including over 150 patients.

**TABLE 1:** The main features of eligible studies in this review.

Study/publication year	Endpoints	ECOG	Sex			Sample size		Intervention		Clinical status	Allocation	Masking	Intervention model	Total time	Follow up	Location
			M	F	Age	Treatment	Control	Treatment	Control							
Feldman et al. 2011 [33]	Primary endpoint: Determine the maximum‐tolerated dose, dose‐limiting toxicities, and pharmacokinetics of CPX‐351 Secondary endpoint: Evaluate safety and determine whether complete remissions can be achieved.	0‐2	31	17	Median age 62 years (range, 23–81)	48	0	CPX‐351	NA	Phase 1	Nonrandomized	None (open label)	Single group	24 months	Ranged from 2 to 4 weeks post‐induction to 12 months or longer for duration of response assessment	United States
Lancet et al. 2014 [21]	Primary endpoint: complete remission and in complete remission Secondary endpoint: EFS and OS	0–2	78	48	69–60 years *N* (77) 70–75 years *N* (49)	85	41	CPX‐351	7 + 3 regimen	Phase 2	Randomized	None (open label)	Parallel‐arm	24 months	Twenty‐four months	United States and Canada
Cortes et al. 2015 [22]	Primary endpoint: survival at 12 months Secondary endpoint: EFS and OS	0–2	57	68	60–18 years *N* (103) 61‐65 years *N* (22)	81	44	CPX‐351	Salvage therapy (Various cytarabine‐based regimens): cytarabine (97.7%) and anthracycline (77.3%), usually with additional agents (79.4%), such as etoposide (54.5%) or gemtuzumab ozogamicin (18.2%)	Phase 2	Randomized	None (open label)	Parallel‐arm	One year	Median 12 months	United States and Canada
Krauss et al. 2019 [23]	Primary endpoints: Overall survival and safety Secondary endpoints: CR	0–2	190	119	Median years 68	153	156	CPX‐351	7 + 3 regimen	Phase 3	Randomized	None (open label)	Parallel‐arm	Five years	Median 9.6 months	United States and Canada
Walter et al. 2018 [24]	Primary endpoints: Efficacy (CR rate) Secondary endpoint: Safety	1–3	31	17	Median 70.6	38	10	CPX‐351 32 units/m^2^	CPX‐351 64 units/m^2^	Phase 2	Randomized	None (open label)	Parallel‐arm	NA	NA	United States
Lancet et al. 2018 [4]	Primary endpoints: Overall survival Secondary endpoints: Remission rate, remission duration, event‐free survival, safety	0–2	190	119	Median 67.8 years	153	156	CPX‐351	7 + 3 regimen	Phase 3	Randomized	None (open label)	Parallel‐arm	23 months	Median 20.7 months	United States and Canada
Kolitz et al. 2019 [25]	Primary endpoint: OS Secondary endpoint: (CR, CR + Cri)	0–2	48	31	69–60 years *N* (50), 70–75 years *n* (31)	49	31	CPX‐351	7 + 3 regimen	Phase 3	Randomized	None (open label)	Parallel‐arm	NA	The follow up after intervention took median 9.7 months	United States and Canada
Lin et al. 2019 [32]	Primary endpoint: Effect of CPX‐351 on cardiac repolarization as measured by change from baseline in QTcF Secondary endpoint: Characterize pharmacokinetics of CPX‐351 components. Assess relationship between drug concentrations and QTc intervals	0–2	14	12	Median age 67 years	26	0	CPX‐351	NA	Phase 2	Non‐randomized	None (open label)	Single group	3 months	2 months	United States
Roboz et al. 2020 [19]	Primary endpoints: safety Secondary endpoints: CR or CRi	0–2	25	27	55–65 years *N* (12) > 65–75 years *N* (40)	52	0	CPX‐351	NA	Phase 4	Nonrandomized	None (open label)	Single group	336 days	35.5 days (range: 21–245)	United States
Guolo et al. 2020 [31]	Primary endpoint: safety Secondary endpoint: CR or Cri, OS	0–4	39	32	< 70 Years *N* (51) > 70 Years *N* (20)	71	0	CPX‐351	NA	Compassionate use program	Nonrandomized	None (open label)	Single group	1 year and 1 month	A median follow‐up of 11 months	Italy
Issa et al. 2020 [26]	Primary endpoints: CR/CRi rate Secondary endpoints: EFS, OS, safety	0–3	37	19	Median: 69 (range 55–84)	16	40	CPX‐351 50 units/m^2^	CPX‐351 75 units/m^2^ 100 units/m^2^	Phase 2	Randomized	None (open label)	Parallel‐arm	NA	Median 27.8 months	United States
Lin et al. 2021 [27]	Primary endpoints: OS Secondary endpoints: remission rate, safety	0–2	70	55	65‐60 years *n* (85), 70–75 years *n* (40)	73	52	CPX‐351	7 + 3 regimen	Phase 3	Randomized	None (open label)	Parallel‐arm	NA	Between 2 and 4 years	United States
Donnette et al. 2021 [30]	Primary endpoint: estimate individual PK parameters and derive cytarabine systemic exposure parameters. Secondary endpoint: to estimate the accumulation of liposomal cytarabine in bone marrow and to tentatively associate PK parameters of liposomal cytarabine with clinical outcome, toxicity	NR	3	6	64 ± 12 (38–77) Years	9	0	CPX‐351	NA	Phase 1	Nonrandomized	None (open label)	Single group	NA	NA	France
Othman et al. 2023 [28]	Primary endpoint: OS Secondary endpoint: Exploratory analysis of molecularly defined subgroups	0–2	108	79	Median 57 years	105	82	CPX‐351	FLAG‐Ida comprised fludarabine 30 mg/m2 IV on days 2 to 6 inclusive cytarabine 2 g/m^2^ over 4 h starting 4 h after fludarabine on days 2–6	Phase 3	Randomized	None (open label)	Parallel‐arm	4 years	The follow up time after intervention took about (median): 54 months	United Kingdom

*Note:* Total timeis the entire time taken for the study to be conducted, while the follow up time is the time taken to follow up patients and control after intervention takes place.

Abbreviations: AML: acute myeloid leukemia; CDA: cytidine deaminase activity; CPX‐351: cytarabine and daunorubicin Liposome; CR: complete remission; Cri: complete response with incomplete count recovery; ECOG: Eastern Cooperative Oncology Group; F: female; FLAG‐Ida: combination of (fludarabine, cytarabine, granulocyte‐colony stimulating factor and idarubicin); HSCT: hematopoietic stem cell transplantation; IV: intravenous; M: male; MDS: Myelodysplastic syndrome; N: number; NA: total time taken for the study is not available; OS: overall survival; PK: Pharmacokinetics; QTcF: Fridericia's corrected QT interval; RFS: relapse‐free survival; sAML: secondary AML; tAML: therapy‐related AML.

Follow‐up times varied substantially across studies, ranging from under 3 months to over 5 years. Primary endpoints predominantly focused on measures of treatment response, such as CR rates, as well as long‐term outcomes, such as OS. Secondary endpoints provided additional data on clinical outcomes, safety, pharmacokinetics, molecular characteristics, and quality of life measures. The age of recruited patients ranged from young adults to older adults, with both newly diagnosed and relapsed AML. These diverse studies have collected evidence on the safety, efficacy, optimal dosing, and patient subgroups that benefit the most from CPX‐351 compared to standard therapies.

### 3.3 Safety

Febrile neutropenia demonstrated a broad range of incidences, occurring in 11%–69% of cases across the studies. For instance, in the study by Lancet et al., 52 patients in the treatment group experienced febrile neutropenia compared to 20 in the control group, while the study by Cortes et al. reported 43 cases in the treatment group versus 14 in the control group [21, 22]. Other infections, such as pneumonia (1%–24%), bacteremia (2%–15%), and sepsis (1%–23%), were also observed. Specifically, pneumonia was reported in 11 treatment group patients compared to 2 in the control group (Cortes et al.), bacteremia in 30 treatment group patients versus 8 in the control group (Lancet et al.), and sepsis in 2 treatment group patients compared to 1 in the control group (Walter et al.) [21, 22, 24]. Additional adverse events (AEs) were noted, including diarrhea/colitis in 69 treatment group patients compared to 100 in the control group (Krauss et al.) and constipation in 36 treatment group patients versus 24 in the control group (Lin et al.) [23, 27]. Cardiac arrhythmias were observed in 46 treatment group patients compared to 41 in the control group, and musculoskeletal pain was reported in 58 versus 52 patients, respectively, both in the Krauss et al. study [23]. Abdominal pain was also documented in 51 treatment group patients compared to 45 in the control group in the same study. Beyond physical symptoms, psychological and systemic adverse events were also evaluated. Anxiety occurred in 21 treatment group patients versus 16 in the control group (Krauss et al.), while delirium was reported in 24 treatment group patients compared to 33 in the control group. Sleep disorders were noted in 38 treatment group patients versus 42 in the control group, both in the Krauss et al. study [23] (Table 2).

**TABLE 2:** Adverse events reported in the included studies.

Study	Intervention model	Intervention		Sample size		Adverse events			
		Treatment group	Control group	Treatment group	Control group	Treatment group	(*n*)	Control group	(*n*)
Feldman, 2011 [33]	Single group	CPX‐351 doses from 3 to 134 units/m^2^	—	48	0	**Grade 3**		—	—
						Mucositis Vomiting Skin rash Cardiac LVEF Bilirubin ALT/AST elevation	1 1 3 2 1 2		
Lancet, 2014 [21]	Parallel‐arm	CPX‐351	7 + 3 regimen	85	41	**Grade 3**		**Grade 3**	
						Febrile neutropenia bacteremia Pneumonia Hypokalemia Sepsis Fungal infection Diarrhea Fatigue Acute renal failure Urinary tract infection Syncope Hypoxia Mental status changes Rash renal failure	52 30 12 12 4 11 8 7 5 6 6 5 4 7	Febrile neutropenia bacteremia Pneumonia Hypokalemia Sepsis Fungal infection Neutropenia Acute renal failure Urinary tract infection Mental status changes Renal failure	20 8 7 5 2 1 3 2 3 3 3
						**Grade 4**	2	**Grade 4**	
						Febrile neutropenia hypokalemia Sepsis Fungal infection Neutropenia Thrombocytopenia Acute renal failure Renal failure	2 1 4 1 6 7 3	Febrile neutropenia thrombocytopenia Renal failure	1 3 4 1
						**Grade 5**	1	**Grade 5**	1
						Pneumonia Sepsis	1 2	Pneumonia sepsis	3
Cortes, 2015 [22]	Parallel‐arm	CPX‐351	Salvage therapy (Various cytarabine‐based regimens): cytarabine (97.7%) and anthracycline (77.3%), usually with additional agents (79.4%), such as etoposide (54.5%) or gemtuzumab ozogamicin (18.2%)	81	44	**Grade 3**		**Grade 3**	
						Febrile neutropenia Bacteremia Pneumonia Hypokalemia Sepsis Fatigue Acute renal failure Urinary tract infection Rash Pyrexia Malignant neoplasm progressive Cellulitis hypertension	43 21 11 7 2 11 4 5 9 7 2 4	Febrile neutropenia Bacteremia Pneumonia Hypokalemia Sepsis Acute renal failure Urinary tract infection Cellulitis Hypertension	14 17 2 3 1 3 5 2 3
						**Grade 4**	4	**Grade 4**	
						Febrile neutropenia bacteremia Pneumonia Sepsis Fatigue Malignant neoplasm Progressive	1 3 3 5 1 1	Febrile neutropenia Bacteremia Sepsis	1 1 2
						**Grade 5**		**Grade 5**	
						Pneumonia sepsis Malignant neoplasm Progressive	4 4 2	Bacteremia Pneumonia Malignant neoplasm progressive	1 2 2
Krauss, 2019 [23]	Parallel‐arm	CPX‐351	7 + 3 regimen	153	156	**All grades**		**All grades**	
						Hemorrhage Febrile neutropenia Rash Edema Nausea Diarrhea/colitis Mucositis Constipation Musculoskeletal pain Abdominal pain Cough Headache Dyspnea Fatigue Arrhythmia Decreased appetite Pneumonia excluding fungal. Sleep disorder Bacteremia excluding sepsis. Vomiting Chills Hypotension Non conduction cardiotoxicity Dizziness Fungal infection Hypertension Hypoxia URTI excluding fungal Chest pain. Pyrexia Catheter device injection site reaction Delirium Pleural effusion Anxiety Pruritis Sepsis excluding fungal Hemorrhoids Petechiae Renal insufficiency Transfusion reaction Visual impairment except bleeding	107 104 82 78 72 69 67 61 58 51 51 51 49 49 46 44 39 38 37 37 35 30 31 27 27 28 28 28 26 26 24 24 24 21 23 17 16 17 17 16 16	Hemorrhage Febrile neutropenia Rash Edema Nausea Diarrhea/colitis Mucositis Constipation Musculoskeletal pain Abdominal pain Cough Headache Dyspnea Fatigue Arrhythmia Decreased appetite Pneumonia excluding fungal. Sleep disorder Bacteremia excluding sepsis. Vomiting Chills Hypotension Non conduction cardiotoxicity Dizziness Fungal infection Hypertension Hypoxia URTI excluding fungal Chest pain Pyrexia Catheter device injection site reaction Delirium Pleural effusion Anxiety Pruritis Sepsis excluding fungal Hemorrhoids Petechiae Renal insufficiency Transfusion reaction Visual impairment except bleeding	74 103 55 90 79 100 69 57 52 45 34 36 51 58 41 57 35 42 37 33 38 32 27 26 19 22 31 19 22 23 15 33 25 16 14 20 12 17 17 16 8
Walter, 2018 [24]	Parallel‐arm	CPX‐351 32 units/m^2^	CPX‐351 64 units/m^2^	38	10	**TEAE grade 3–5**		**TEAE grade 3–5**	
						Catheter related infection Lung infection Neutropenic fever Sepsis Cardiac arrest Mucositis AKI ALT increase AST increase Dehydration Tumor lysis Intracranial hemorrhage Bronchopulmonary hemorrhage DVT	2 3 7 2 1 2 4 1 1 1 1 1 1 1	Catheter related infection Cellulitis lung infection Neutropenic fever Sepsis Atrial tachycardia Tachycardia Mucositis AKI Tumor lysis Hypoxia ARDS Atelectasis Respiratory failure DVT Leg pain Urinary retention	1 1 3 5 1 1 1 2 2 1 2 1 1 1 1 1 1
Lancet, 2018 [4]	Parallel‐arm	CPX‐351	7 + 3 regimen	153	156	**Grade 3–5**		**Grade 3–5**	
						Febrile neutropenia Pneumonia Hypoxia Bacteremia Respiratory failure Sepsis Hypertension Decreased ejection fraction	104 30 20 11 10 8 12 2	Febrile neutropenia Pneumonia Hypoxia Bacteremia Respiratory failure Sepsis Hypertension Decreased ejection fraction	107 22 23 7 5 11 8 5
Kolitz, 2019 [25]	Parallel‐arm	CPX‐351	7 + 3 regimen	49	31	**Any TEAE**		**Any TEAE**	
						Febrile neutropenia Nausea Constipation Fatigue Pyrexia Epistaxis Diarrhea Peripheral edema Decreased appetite Chills Rash rthralgiaa Dizziness Headache Oropharyngeal pain Skin lesion Mucosal inflammation	62 11 12 12 8 8 6 8 8 10 4 6 7 4 3 1 3	Febrile neutropenia Nausea Constipation Fatigue Pyrexia Epistaxis Diarrhea Peripheral edema Decreases appetite Chills Rash Dizziness Headache Oropharyngeal pain Skin lesion Atrial fibrillation rhinorrhea Mucosal inflammation	41 9 10 11 4 2 10 8 4 4 3 6 5 5 4 4 3 1
						**Grade 3–5 TEAE**		**Grade 3–5 TEAE**	
						Febrile neutropenia	16	Febrile neutropenia	9
						**Serious TEAE**		**Serious TEAE**	
						Febrile neutropenia	8	Febrile neutropenia	7
Lin, 2019 [32]	Single group	CPX‐351	—	26	0	**Grade 1–3**		—	—
						Febrile neutropenia Fatigue Nausea Decreased appetite Diarrhea	19 14 14 12 12		
Roboz, 2020 [19]	Single group	CPX‐351	—	52	0	**TEAEs any grade**		—	—
						Febrile neutropenia Hypoxia Pneumonia Hypertension	40 12 7 7		
						**Grade 3**			
						Epistaxis Purpura Infection Lung infection Sepsis Staphylococcal infection	1 1 4 4 3 2		
						**Grade 4**			
						Subdural hematoma	1		
						**Grade 5**			
						Intracranial hemorrhage	1		
Guolo, 2020 [31]	Single group	CPX‐351	—	71	0	**Grade** > **1 AEs**		—	—
						Fever of unknown origin sepsis Pneumonia Pneumocystis jirovecii‐related pneumonia Invasive fungal infection mucositis Diffuse skin rash Alopecia	29 23 11 3 3 7 26 4		
Issa, 2020 [26]	Parallel‐arm	CPX‐351 50 units/m^2^	CPX‐351 75 units/m^2^/ CPX‐35,1100 units/m^2^	16	40	**Grade 3**		**Grade 3**	
						Febrile neutropenia Skin infection GIT hemorrhage Edema limbs	1 1 1 1 2 2	Febrile neutropenia Pneumonia Skin infection ALT/AST elevation Bilirubin increased ACS Pancreatitis joint effusion Edema limbs hemorrhageGIT Hypotension Enterocolitis	18 13 2 2 2 1 1 1 1 1 1 1
						**Grade 4**		**Grade 4**	
						Sepsis	2	Sepsis Respiratory failure hypotension Pleural effusion Intracranial hemorrhage	7 2 2 1 1
						**Grade 5**		**Grade 5**	
						Pneumonia	2	Respiratory failure Multiorgan failure Sepsis	3 2 1
Lin, 2021 [27]	Parallel‐arm	CPX‐351	7 + 3 regimen	73	52	**Any TEAE**		**Any TEAE**	
						Febrile neutropenia Nausea Constipation Peripheral edema Decreased appetite Fatigue Rash Diarrhea Dyspnea epistaxis Chills Cough Headache Hypotension Dizziness Mucosal Inflammation Pyrexia Insomnia Pleural effusion	60 38 36 32 30 29 29 28 28 25 23 23 22 20 19 19 17 16 12	Febrile neutropenia Nausea Constipation Peripheral edema Decreased appetite Fatigue Rash Diarrhea Dyspnea Epistaxis Chills Cough Headache Hypotension Dizziness Mucosal Inflammation pyrexia Insomnia Pleural effusion	40 28 24 29 23 26 15 38 13 11 20 15 18 13 19 9 14 13 15
						**Any Serious TEAE**		**Any Serious TEAE**	
						Febrile neutropenia Acute respiratory failure Ejection fraction decreased Sepsis Pneumonia Pulmonary edema	11 5 4 4 2 1	febrile neutropenia Acute respiratory failure Ejection fraction decreased sepsis Pneumonia Pulmonary edema	6 1 2 2 3 3
Donnet, 2021 [30]	Single group	CPX‐351	—	9	0	**Grade 3–4**		—	—
						Constipation Toxic death (colonic obstruction) Nausea Anorexia Toxidermia Rectal bleeding Hemorrhoid febrile Neutropenia	1 1 1 1 2 1 1 9		

Abbreviations: ACS: acute coronary syndrome; AEs: adverse events; AKI: acute kidney injury; ALT: Alanine transaminase; ARDS: acute respiratory distress syndrome; AST: Aspartate aminotransferase; DVT: deep vein thrombosis; GIT: gastrointestinal tract; LVEF: left ventricular ejection fraction; TEAE: treatment‐emergent adverse events; URTI: upper respiratory tract infection.

Among the array of adverse events (AEs) reported, hemorrhage emerged as a notable concern, with 107 patients in the treatment group compared to 74 patients in the control group in the study by Krauss et al. The frequency of hemorrhage varied, ranging from zero occurrences to 13% in the treatment group [23].

Another significant adverse event, febrile neutropenia, exhibited a wide range of occurrences, from 11% to 69% across the studies. For example, febrile neutropenia occurred in 52 patients in the treatment group compared to 20 patients in the control group in the study by Lancet et al., and in 43 patients in the treatment group compared to 14 patients in the control group in the study by Cortes et al. [22].

Pneumonia, bacteremia, and sepsis were also observed among the reported adverse events, with incidence rates ranging from 1% to 24%, 2% to 15%, and 1% to 23%, respectively. For example, in the study by Cortes et al., pneumonia was documented in 11 patients in the treatment group compared to 2 in the control group. Similarly, bacteremia was reported in 30 patients in the treatment group versus 8 in the control group in the study by Lancet et al., while sepsis was noted in 2 patients in the treatment group compared to 1 in the control group in the study by Walter et al. [21, 22, 23, 24].

Other additional AEs include diarrhea/colitis, which was reported in 69 patients in the treatment group compared to 100 patients in the control group in the study by Krauss et al., and constipation, which occurred in 36 patients in the treatment group compared to 24 patients in the control group in the study by Lin et al. [23, 32].

Arrhythmia was reported in 46 patients in the treatment group compared to 41 in the control group, as documented in the study by Krauss et al. Musculoskeletal pain was observed in 58 patients in the treatment group versus 52 in the control group, while abdominal pain was reported in 51 treatment group patients compared to 45 in the control group in the same study. In addition to physical symptoms, psychological and systemic effects were also evaluated. Anxiety was reported in 21 patients in the treatment group compared to 16 in the control group (Krauss et al.). Delirium occurred in 24 treatment group patients versus 33 in the control group, while sleep disorders were noted in 38 treatment group patients compared to 42 in the control group, both findings from the Krauss et al. study [23, 27].

Cardiovascular and respiratory complications, such as hypertension as reported among *n* = 12 in the treatment group compared to *n* = 8 in the control group in the Lancet et al. study, non‐conduction cardiotoxicity as reported among *n* = 31 in the treatment group compared to *n* = 27 in the control group in the Krauss et al. study, and upper respiratory tract infections (URTI), excluding fungal cases, were observed as reported among *n* = 28 in the treatment group compared to *n* = 19 in the control group in the Krauss et al. study. Additionally, various localized reactions, such as catheter device injection site reactions as reported among *n* = 24 in the treatment group compared to *n* = 15 in the control group in the Krauss et al. study, pleural effusion as reported among *n* = 24 in the treatment group compared to *n* = 25 in the control group in the Krauss et al. study, and pruritus as reported among *n* = 23 in the treatment group compared to *n* = 14 in the control group in the Krauss et al. study [4, 23].

**FIGURE 3:**
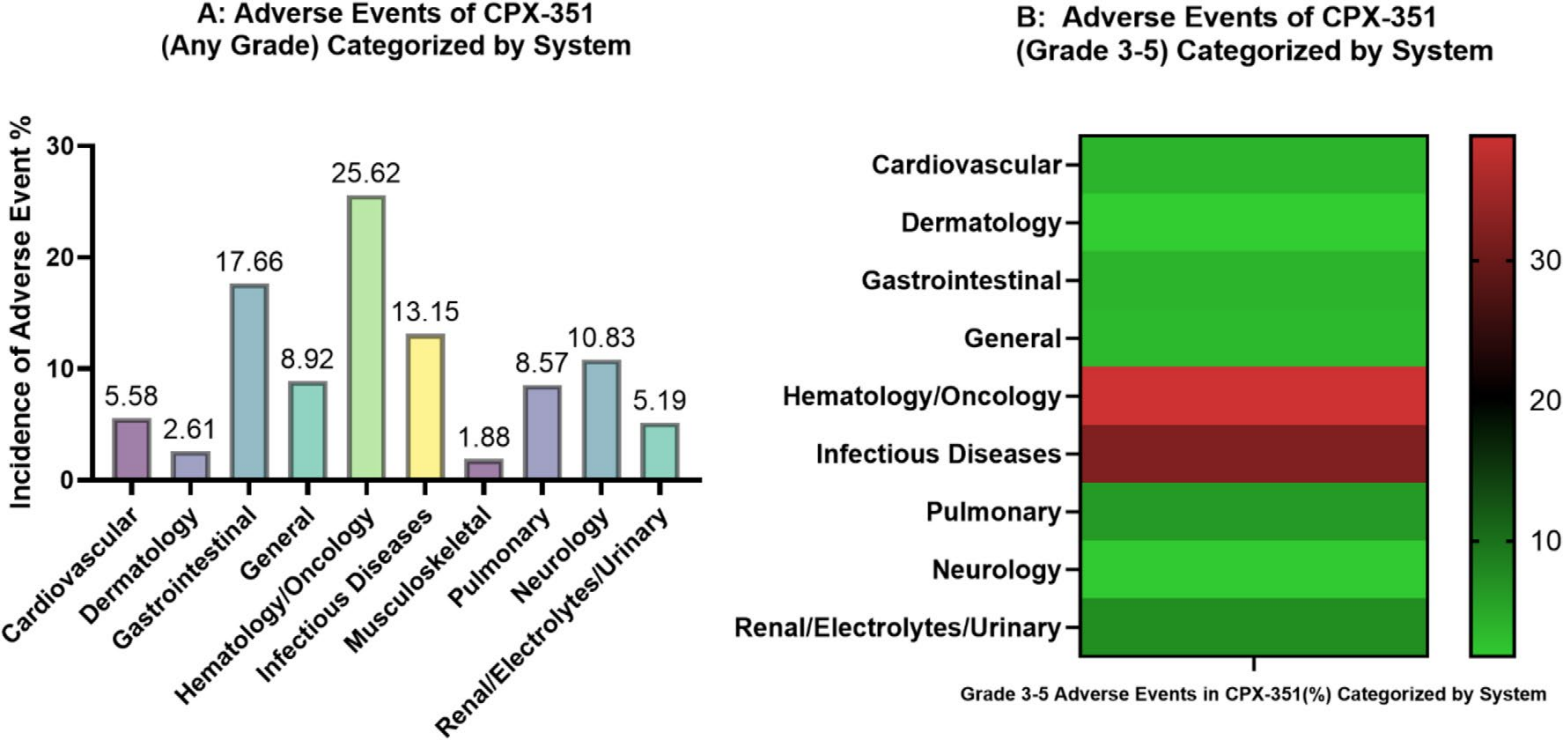
Distribution and Severity of CPX‐351 Adverse Events Across Different Organ Systems for any grade (A) and for grade 3‐5 (B). (A) Frequency of CPX‐351 adverse events (AEs), with hematology/oncology (25.48%), gastrointestinal (16.55%), and infectious disease (15.09%) AEs most common. (B) Heatmap of severe (Grades 3–5) AEs, highlighting hematology/oncology (40%) and infectious disease (32%) as the most impactful.

The most frequently reported adverse events (AEs) were hematology/oncology‐related (*n* = 873, 25.48%), followed by gastrointestinal AEs (*n* = 567, 16.55%), infectious disease AEs (*n* = 517, 15.09%), and neurological AEs (*n* = 369, 10.77%) (Figure 2A). Among kidney‐related AEs, peripheral edema was the most common, with 119 cases, followed by acute kidney injury, reported in 33 cases. Skin rash was the predominant skin‐related AE, occurring in 134 instances. Cardiovascular AEs associated with CPX‐351 included hypertension (51 cases), hypotension (50 cases), and arrhythmia (46 cases). Nausea emerged as the most frequently reported gastrointestinal AE, with 136 events. Pulmonary AEs, such as dyspnea, hypoxemia, and cough, were also commonly observed. Additional details on these AEs are provided in Figure 3A,B.

### 3.4 Grade 3–5 Adverse Events

Grade 3–5 adverse events were reported across nine systems, with the most severe affecting hematology/oncology (282 events, 40%), followed by infectious diseases (231 events, 32%), renal/electrolytes/urinary (53 events, 7%), and pulmonary systems (46 events, 6%) (Figure 3B). Febrile neutropenia of any grade was documented in five studies, accounting for a total of 296 cases. Among these, Grade 3–4 febrile neutropenia was reported in seven studies, totaling 266 events.

### 3.5 Efficacy

Ten out of the 14 clinical trials reported overall survival (OS) outcomes, involving 824 patients who received the CPX‐351 regimen [4, 21, 22, 23, 24, 25, 26, 27]. The highest median OS was 25.4 months with a follow‐up duration of 2–4 years [25], while the lowest median OS was 3 months [24].

Five studies reported event‐free survival (EFS), with the highest median EFS being 6.5 months and a median follow‐up duration of 24 months [21]. All clinical trials reported complete remission (CR), with 423 of 959 (44.1%) patients achieving CR following CPX‐351 treatment. Additionally, 91 of 806 (11.3%) patients achieved CRi. The efficacy data from these studies are summarized in Table 3.

**TABLE 3:** Efficacy data of studies included.

Clinical trial	Follow up	Intervention model	Intervention		Sample size		CPX‐351 treatment stage	Patient status	Overall survival		Event free survival		Complete remission		CRi		Partial response		Relapse free survival	
			Study arm	Control arm	Study arm	Control arm			Study arm	Control arm	Study arm	Control arm	Study arm	Control arm	Study arm	Control arm	Study arm	Control arm	Study arm	Control arm
Feldman et al. 2011 (phase 1) [33]	Ranged from 2 to 4 weeks post‐induction to 12 months or longer for duration of response assessment	Single group	CPX‐351	NA	48	0	Induction regimen	Relapsed and Refractory	NA	NA	NA	NA	9	NA	1	NA	NA	NA	NA	NA
Lancet et al. 2014 (Phase2) [21]	Median Twenty‐four months	Parallel‐arm	CPX‐351	7 + 3 regimen	85	41	Induction and consolidation regimen	Untreated AML	Median OS 14.7 months	Median OS 12.9 months	EFS median (6.5 months)	EFS median two months	41	20	15	1	NA	NA	NA	NA
Cortes et al. 2015 (phase 2) [22]	Median 12 months	Parallel‐arm	CPX‐351	Salvage therapy (Various cytarabine‐based regimens): cytarabine (97.7%) and anthracycline (77.3%) usually with additional agents (79.4%), such as etoposide (54.5%) or gemtuzumab ozogamicin (18.2%)	81	44	induction and consolidation regimen	First relapse AML	OS median (8.5 months)	OS median (6.3 months)	Median (4 months)	Median 1.5 months	30	14	10	4	NA	NA	NA	NA
Krauss et al. 2019 (phase 3) [23]	Median 9.6 months	Parallel‐arm	CPX‐351	7 + 3 regimen	153	156	Induction and consolidation regimen	Newly diagnosed t‐AML	Median 9.6 months	Median (5.9 months)	NA	NA	58	41	NA	NA	NA	NA	NA	NA
Walter et al.2018 (Phase2) [24]	NA	Parallel‐arm	CPX‐351 32 units/m2	CPX‐351 64 units/m2	38	10	Induction regimen	Untreated AML	Median 3 months	Median 6 months	NA	NA	8	1	1	1	Median 7 months	NA	NA	NA
Lancet et al. 2018 (Phase 3) [4]	Median 20.7 months	Parallel‐arm	CPX‐351	7 + 3 regimen	153	156	induction and consolidation regimen	Newly Diagnosed	9.56 months	5.95 months	2.53 months	1.31 months	57	40	16	12	NA	NA	NA	NA
Kolitz et al. 2019 (Phase 3) [25]	Median 9.7 months	Parallel‐arm	CPX‐351	7 + 3 regimen	49	31	Consolidation and induction regimen	High‐risk/secondary AML	Median 25.43 months	Median 8.53 month	NA	NA	40	30	6	2	NA	NA	NA	NA
Lin et al. 2019 (Phase 2) [32]	Median 60 days	Single group	CPX‐351	NA	26	0	Induction and consolidation regimen	Newly diagnosed or relapsed/refractory AML	NA	NA	NA	NA	8	NA	5	NA	NA	NA	NA	NA
Roboz et al. 2020 (Phase 4) [19]	Median 53.5 days (range: 21–245	Single group	CPX‐351	NA	52	0	Induction and consolidation regimen	High‐risk or secondary AML	NA	NA	NA	NA	15	NA	8	NA	NA	NA	NA	NA
Guolo et al. 2020 (Compassionate use program) [31]	A median follow‐up of 11 months	Single group	CPX‐351	NA	71	0	Induction and consolidation regimen	Newly diagnosed sAML or tAML	Twelve‐months OS was 68% (median not reached)	NA	NA	NA	38	NA	8	NA	6	NA	NA	NA
Issa et al. 2020 (Phase 2) [26]	Median 27.8 months	Parallel‐arm	CPX‐35150 units/m^2^	CPX‐351 75 units/m^2^/CPX‐351100 units/m^2^	16	40	Induction regimen	Newly diagnosed	Median: 4.3 months	75 units/m^2^: 8.6 months 100 units/m^2^: 6.2 months	Median 1.2 months	75 units/m^2^: median EFS 2.0 months 100 units/m^2^: median EFS 3.3 months	3	75 units/m^2^:6 100 units/m2:7	0	75 units/m^2^: 3 100 units/m2: 0	NA	NA	NA	NA
Lin et al. 2021 (Phase 3) [27]	Between 2 and 4 years	Parallel‐arm	CPX‐351	7 + 3 regimen	73	52	Induction regimen	High‐risk/secondary AML	Median 25.4 months	Median 10.4 months	NA	NA	57	40	16	12	NA	NA	Median 11.2 months	Median 8.22 months
Donnette et al. 2021 (Phase 1) [30]	NA	Single group	CPX‐351	NA	9	0	Induction regimen	Newly diagnosed	NA	NA	NA	NA	5	NA	1	NA	1	NA	NA	NA
Othman et al. 2023 (Phase 3) [28]	(Median): 54 months	Parallel‐arm	CPX‐351	FLAG‐Ida comprised fludarabine 30 mg/m^2^ IV on days 2–6 inclusive, cytarabine 2 g/m^2^ over 4 h starting 4 h after fludarabine on days 2–6	105	82	Induction and post‐remission regimen	Adverse karyotype AML	Median 13.3 months	Median 11.4 months	Not significantly different between the CPX‐351 and Control (FLAG‐Ida) groups. Hazard ratio for EFS (CPX‐351 vs. Control): HR 0.97 (95% CI 0.69‐1.37), *p* = 0.86		54	43	4	7	NA	NA	Median 22.1 months	Median 8.35 months

Abbreviations: AML: Acute Myeloid Leukemia; sAML: secondary AML; tAML: therapy‐related AML.

Across multiple clinical trials, CPX‐351 demonstrated efficacy in treating acute myeloid leukemia (AML), with variable response rates depending on the disease stage. In single‐arm studies of relapsed, refractory, or high‐risk AML, CPX‐351 achieved CR rates ranging from 19% to 82%, with a median OS of 6.3–25.4 months.

In randomized controlled trials comparing CPX‐351 to conventional chemotherapy regimens like 7 + 3, CPX‐351 showed improved response rates and survival outcomes. For example, OS was 14.7 months for CPX‐351 versus 12.9 months for 7 + 3 [28]. In newly diagnosed AML, CPX‐351 led to CR rates of 19%–54%, compared to 14%–40% for the control arm. The median OS was 4.3–9.6 months for CPX‐351, compared to 5.9–8.6 months for the control arm.

In relapsed or refractory AML, CPX‐351 showed superior CR rates (37% vs. 31.8%) and OS (8.5 months vs. 6.3 months) when compared to salvage chemotherapy. In consolidation therapy for high‐risk/secondary AML, CPX‐351 was associated with longer relapse‐free survival (RFS) (11.2 and 22.1 months vs. 8.22 and 8.35 months), higher CR rates (57% vs. 40%), and longer OS (25.4 vs. 10.4 months) compared to the 7 + 3 regimen.

## 4 Risk of Bias (ROB) Assessment

The risk of bias (ROB) assessment was conducted for nine randomized controlled trials (RCTs) using the Cochrane risk‐of‐bias tool for randomized trials (RoB 2). This evaluation was initially performed by the first author and subsequently reviewed by the corresponding author. Among the RCTs, one study was classified as having a low ROB (Lin et al.) [27], while two studies demonstrated high ROB in the overall assessment (Lancet et al.; Cortes et al.) [21, 22] (Table 4). For the five non‐randomized studies of interventions (NRSI), the ROBINS‐I tool was utilized (Feldman et al.; Lin et al.; Roboz et al.; Guolo et al.; Donnette et al.) [19, 30, 31, 32, 33]. Among these, one study (Roboz et al.) [19] was identified as having a critical ROB, while three studies were rated as having a serious ROB (Feldman et al.; Guolo et al.; Donnette et al.) (Table 5) [30, 31, 33].

**TABLE 4:** Review author judgments about risk of bias criteria (RoB2) for randomized clinical trials.

Study	Selection bias	Performance bias	Detection bias	Attrition bias	Reporting bias	Other bias	Overall risk of bias
Lancet 2014 [21]	Low	Some concerns	High	Low	Some concerns	Low	High
Cortes 2015 [22]	Some concerns	Some concerns	High	Low	Some concerns	Low	High
Krauss 2019 [23]	Low	Some concerns	Low	Low	Low	Low	Some concerns
Walter 2018 [24]	Low	Some concerns	Low	Low	Low	Low	Some concerns
Lancet 2018 [4]	Low	Some concerns	Low	Low	Low	Low	Some concerns
Kolitz 2019 [25]	Low	Some concerns	Low	Low	Low	Low	Some concerns
Issa 2020 [26]	Low	Some concerns	Low	Low	Low	Low	Some concerns
Lin 2021 [27]	Low	Low	Low	Low	Low	Low	Low
Othman 2023 [28]	Low	Low	Low	Low	Low	Some concerns	Some concerns

**TABLE 5:** Review authors' judgments about each risk of bias criteria ROBINS‐1 for non‐randomized studies.

Study	Confounding	Selection bias	Misclassification bias	Performance bias	Attrition bias	Detection bias	Reporting bias	Overall risk of bias
Feldman 2011 [33]	Serious	Low	Low	Low	Low	Moderate	Low	Serious
Lin 2019 [32]	Moderate	Low	Low	Low	Low	Moderate	Low	Moderate
Roboz 2020 [19]	Critical	Low	Low	No Information	Low	Low	Low	Critical
Guolo 2020 [31]	Serious	Low	Low	No Information	Low	Low	Moderate	Serious
Donnette 2021 [30]	Serious	Low	Low	Low	Low	Moderate	Moderate	Serious

## 5 Discussion

This systematic review consolidates evidence from 14 clinical trials involving over 800 patients, underscoring CPX‐351 as a significant advancement in AML treatment. Compared to conventional 7 + 3 chemotherapy regimens, CPX‐351 demonstrates superior safety and efficacy, with improved complete remission (CR) rates, relapse‐free survival (RFS), and overall survival (OS), particularly in high‐risk and secondary AML cases. These findings align with phases 2 and 3 trials conducted by Lancet et al. [21] and Lin et al. [27], which provided the basis for CPX‐351's FDA approval. Notably, CPX‐351's targeted liposomal delivery system enhances antileukemic activity while reducing systemic toxicity, offering a more favorable toxicity profile than traditional chemotherapies [5, 15]. Despite these promising outcomes, certain methodological considerations and limitations must be acknowledged. Variations in study designs, endpoints, and reporting across the included trials may have introduced heterogeneity into the analysis. The risk of bias (ROB) assessment identified critical and serious biases in some non‐randomized studies (e.g., Feldman et al. [33], Guolo et al. [31], and Roboz et al. [19]), emphasizing the need for careful interpretation of observational data. In randomized trials, while one study demonstrated a low ROB (Lin et al. [27]), others exhibited high ROB in their overall assessments (Lancet et al. [21] and Cortes et al. [22]). These findings highlight the importance of evaluating bias at both study‐level and outcome‐specific levels, as not all sources of bias influence outcomes to the same degree [34]. Cochrane guidelines emphasize a domain‐based approach to bias assessment, focusing on elements such as allocation, blinding, and data completeness [35, 36]. For example, outcome‐specific biases, such as masking of outcome assessors and handling of missing data, can vary in their impact. In some studies, missing data from patients with better health may have led to an underestimation of treatment benefits, whereas missing data from more severely affected patients could have led to overestimation [34]. Such nuances underscore the complexity of interpreting bias direction and magnitude. Furthermore, the potential interplay of multiple biases within a study necessitates careful consideration of whether biases counteract or amplify one another [34]. The findings of this review also highlight gaps in long‐term data, particularly concerning cumulative toxicity associated with prolonged CPX‐351 use. While the adverse event (AE) profile of CPX‐351 is consistent with expectations for intensive chemotherapy, systemic toxicities such as febrile neutropenia, pneumonia, and sepsis remain significant concerns. Comparative analyses revealed similar AE profiles between CPX‐351 and 7 + 3 chemotherapy, with slightly lower incidences of certain AEs, such as nausea and diarrhea, in the CPX‐351 arm [21, 23]. Future studies should prioritize standardized adverse event reporting to facilitate more robust comparisons across therapeutic regimens. The evolving landscape of AML treatment, including the emergence of novel targeted therapies and combination regimens, necessitates further investigation into CPX‐351's role. Ongoing trials (e.g., NCT04990102 and NCT04230239) exploring its use in post‐remission consolidation and maintenance therapy will provide critical insights [37, 38]. Moreover, head‐to‐head comparisons with newer targeted agents and combination therapies are essential to establish CPX‐351's optimal position in the AML treatment algorithm [6, 36].

### 5.1 Potential Applications

Novel combinations comprising targeted or other immune treatments have lately been researched with the objective of improving the therapeutic outcomes of CPX‐351 [39].

Consistent synergy was also observed when CPX‐351 was combined with FMS‐like tyrosine kinase 3 (FLT3) inhibitors concurrently or with CPX‐351 exposure planned 24 h before FLT3 inhibitor administration. However, pretreatment with quizartinib for 16 h has the capacity to generate a population of cells (50% of the total population) with diminished daunorubicin fluorescence, demonstrating that lasting FLT3 inhibition may lower CPX‐351 absorption. Edwards et al. reported three secondary acute myeloid leukemia (s‐AML) patients who achieved a complete remission with no noteworthy side effects after taking a combination of CPX‐351 with midostaurin (50 mg twice day from days 8 to 21). The total survival time was 4, 12, as well as 17 months, respectively. Ongoing trials are examining the usage of CPX‐351 and the FLT3 inhibitors (NCT04293562, NCT04128748) [40, 41].

An ongoing trial (NCT03672539) is looking into the safety and efficacy of CPX‐351 in combination with gemtuzumab ozogamicin (GO) in patients having resistant/relapsed acute myeloid leukemia (R/R AML) and post‐hypomethylating agent (HMA) failure high‐risk myelodysplastic syndrome (MDS). The induction plan involves CPX‐351 (daunorubicin 44 mg/m^2^ along with cytarabine 100 mg/m^2^) delivered on days 1, 3, and 5, and GO at 3 mg/m^2^ on day 1. Patients who achieve complete remission or complete remission with incomplete count recovery (CR/CRi) may receive up to two consolidation cycles with CPX‐351 (daunorubicin 29 mg/m^2^ along with cytarabine 65 mg/m^2^) on days 1 and 3 and GO at 3 mg/m^2^ on day 1. To date, 24 patients have been included in the research; among them, 75% previously had a bcl2 inhibitor, venetoclax, along with HMAs or chemotherapy. The ORR rate (CR/CRi) was 55%, although no patients had hematopoietic stem cell transplantation (HSCT) due to age and comorbidities. After the median follow‐up period of 2 years, the median overall survival was 5 months, with a median response duration of 7 months. Infections were the most common cause of adverse events, with a 30‐day mortality rate of 8% [42].

Also, CPX‐351 has showed encouraging outcomes when used with venetoclax, a BCL‐2 inhibitor, in both de novo AML and a subset of R/R AML patients who have undergone extensive pretreatment. An ongoing trial has been planned with two expansion cohorts to study efficacy in R/R AML (Cohort A) as well as frontline AML (Cohort B), after which a safety lead‐in phase has been developed to identify a safe dose and schedule in R/R AML [43].

Additionally, being researched is the use of CPX‐351 in conjunction with immune checkpoint inhibitors. Cytarabine has been shown in multiple tests to upregulate the levels of PD‐1 and reduce the levels of CD80 as well as CD86 in murine models of AML. Additionally, it has been shown that blast cells treated with cytarabine in vivo showed an increased sensitivity to cytotoxic T cell‐mediated destruction. Immune checkpoint inhibitors in combination with CPX‐351 may increase the cytotoxic effect and trigger immune surveillance without lessening cumulative myelosuppression [44].

Other trials are ongoing to evaluate the safety and effectiveness of CPX‐351 with Isocitrate dehydrogenase (IDH) 1 and 2 inhibitors (NCT04493164, NCT03825796), hedgehog cascade inhibitor glasdegib (NCT04231851), JAK2 inhibitor ruxolitinib (NCT03878199), and CDK4/6 inhibitor palbociclib (NCT03844997) [16].

New settings in CPX‐351 scenarios are assessing its potential effectiveness and safety in patients with de novo AML who are classified at intermediate risk corresponding to the European LeukemiaNet (ELN) 2017 prognostic categorization. Interestingly, comparing de novo and stringently characterized secondary AMLs emerging after a recorded phase of MDS, the French group discovered a molecular grouping, termed “secondary‐type AML,” defined by mutations in either SRSF2, SF3B1, U2AF1, ZRSR2, ASXL1, EZH2, BCOR, and/or STAG2 genes. Of the de novo AML patients, 33.3% had secondary‐type mutations. Patients over 60 years old with secondary‐type AML, as defined by that 8‐gene molecular signature, had worse outcomes than those without ‘secondary‐type’ mutations when treated with conventional “3 + 7” chemotherapy, which included cytarabine and an anthracycline (ALFA 1200 study). This was particularly evident among patients with ‘moderate risk’ illness according to the European Leukemia Net (ELN) criteria [45].

The incidence of'secondary type' AML mutations rises with age and cytogenetic risk group. Approximately half of de novo AML patients with moderate risk over the age of 50 have such secondary‐type mutations. New therapeutic alternatives are required in patients over the age of 50 with de novo AML categorized as an unfavorable risk, and in those with intermediate risks and secondary‐type mutations. A current study (NCT052605289) will evaluate the rate of minimal residual disease (MRD) negative remissions with CPX‐351 as induction and consolidation therapy compared to intense chemotherapy within a population of non‐myelodysplasia‐related changes acute myeloid leukemias (MRC AMLs) with secondary‐like mutations [16].

Furthermore, the specific mechanism underlying the improvement in outcomes with CPX‐351, particularly in secondary acute myeloid leukemia (sAML) or therapy‐related acute myeloid leukemia (t‐AML), remains unknown. It has long been understood that certain subgroups are not responsive as well to regular induction, but why that liposomal formulation might improve results in these individuals while making no difference in results in other AML subtypes is an issue for additional research. It is unknown if this reflects the molar ratio of cytarabine and daunorubicin, however past trials to explore different dosages of cytarabine did not appear to have a noticeable dose effect [17, 46, 47, 48].

It could indicate the extended exposure offered by the liposomal formulation, selective uptake of this substance or concentration in the marrow, or another as‐yet‐undetermined mechanism. Furthermore, it is unclear how the survival difference observed in the experiment connects to the initial induction therapy decision. In the pooled sAML study, there was about a 15% overall survival benefit at 1 year with CPX‐351 compared with “7 + 3,” more individuals on the CPX‐351 arm were also able to advance to allogeneic transplant, possibly explaining, in particular, this improvement. Extended cytopenias were more prevalent in patients receiving treatment with CPX‐351, which is frequently a cause of induction‐related fatality; the better early mortality may thus represent higher rates of refractory illness in the “7 + 3” individuals. Understanding the reasons of death for every arm may be illustrative [17].

The excitement surrounding CPX‐351, notably in a usually resistant subtype of AML, has prompted plenty of new investigations on this drug in a variety of contexts. Given its action in sAML as well as tAML, there is interest in investigating CPX‐351 in other relevant contexts, such as myelodysplasia, or for use as a maintenance or consolidation drug for high‐risk diseases. Similarly, if the landscape of AML therapy expands to embrace medicines targeting particular mutations, initiatives to add specific FLT3 or Isocitrate Dehydrogenase (IDH) inhibitors to CPX‐351 may provide further advances to existing AML treatment [17].

Furthermore, further results indicate that CPX‐351 may eventually be successful in high‐risk individuals with MDS and other high‐risk de novo AML, particularly those who are considering allogeneic hematopoietic cell transplantation (HCT). Combining CPX‐351 with small‐molecule inhibitors (e.g., midostaurin, enasidenib, and venetoclax) and/or conjugated monoclonal antibodies may boost efficacy in select patient subsets [4].

Future directions involve assessing CPX‐351's drug effectiveness and toxicology in low‐ as well as intermediate‐risk settings, combining it with targeted therapies, better understanding the mechanistic underpinnings of enhanced responses in t‐AML along with AML‐MRC, and conducting a thorough evaluation of dose intensification in high‐risk settings [16].

### 5.2 Summary of ROB Findings

Only one RCT demonstrated a low ROB, while two exhibited a high ROB overall. The remaining six RCTs showed some concerns regarding ROB, particularly in the domains of performance and detection bias. This suggests that the blinding of participants and outcome assessors was not always adequate, potentially introducing bias into the results.

Three of the five non‐randomized studies were deemed to have a serious risk of bias, primarily due to confounding and selection bias inherent in their designs. One study had a critical risk of bias. These findings underscore the limitations of non‐randomized studies in drawing strong causal inferences.

### 5.3 Potential Impact on Outcomes

The identified risk‐of‐bias issues could have materially influenced the reported outcomes. Lack of blinding in some RCTs—most notably the pivotal open‐label Phase III CPX‐351 vs 7+3 trial (ClinicalTrials.gov NCT01696084; Roboz et al. J Clin Oncol 2016)—might have led investigators or participants to favor CPX‐351, resulting in higher complete remission rates (47.7% vs 33.3%; *P* = 0.016) and longer median overall survival (9.56 vs 5.95 months, HR 0.69; 95% CI, 0.52–0.90) than would have been observed under a truly blinded, placebo‐controlled design such as the CALGB 10603/RATIFY trial (NCT00651261). Similarly, inadequate masking could have led to underreporting of adverse events in the CPX‐351 arm if researchers or patients, aware of treatment assignment, were more inclined to attribute side effects—such as febrile neutropenia and pneumonia—to other causes (adverse event rates reported as comparable between arms). The serious risk of confounding in non‐randomized, real‐world analyses (e.g., a retrospective transplant‐outcomes cohort in older adults) raises concerns that differences in baseline characteristics—such as cytogenetic risk and prior therapy—between CPX‐351 and control groups may have driven the observed benefits rather than the liposomal formulation itself. While our review suggests that CPX‐351 offers promising benefits for AML patients, these ROB assessments underscore important limitations. The potential for bias, particularly in non‐randomized settings, complicates definitive conclusions regarding the magnitude of CPX‐351's efficacy and safety advantages.

## 6 Conclusion

This systematic review provides a comprehensive analysis of the evidence indicating that CPX‐351 represents a significant therapeutic advancement for the treatment of AML. Across 14 clinical trials involving over 800 patients, CPX‐351 demonstrated superior efficacy compared to conventional 7 + 3 chemotherapy in diverse AML populations. Notably, CPX‐351 achieved substantially higher complete remission (CR) rates and extended overall survival (OS) by 3.6–5.7 months compared to control arms in cases of newly diagnosed, relapsed, and refractory AML. These benefits were most evident in patients with high‐risk cytogenetics or secondary AML.

In terms of safety, CPX‐351 exhibited an adverse event (AE) profile consistent with intensive chemotherapy regimens. However, larger randomized controlled trials are warranted, as long‐term safety data remain limited—particularly with prolonged or maintenance CPX‐351 treatment, where the cumulative toxicity is not yet fully understood.

## Author Contributions

Conceptualization: Abdulwahab M. Alzahrani and Mohammed A. Alnuhait. Methodology: Abdulwahab M. Alzahrani, Mohammed A. Alnuhait, and Tariq Alqahtani. Validation: Abdulwahab M. Alzahrani, Mohammed A. Alnuhait, and Tariq Alqahtani. Original draft preparation: Abdulwahab M. Alzahrani, Mohammed A. Alnuhait, and Tariq Alqahtani. Review and editing: Abdulwahab M. Alzahrani, Mohammed A. Alnuhait, and Tariq Alqahtani. All authors have read and agreed to the published version of the manuscript.

## Ethics Statement

The authors have nothing to report.

## Consent

The authors have nothing to report.

## Conflicts of Interest

The authors declare no conflicts of interest.

## Data Availability

The authors have nothing to report.
